# AZ31 Magnesium Alloy Roll-Forming Springback Prediction Considering Anisotropic and Asymmetric Properties

**DOI:** 10.3390/ma18133111

**Published:** 2025-07-01

**Authors:** Yu Yan, Hanzhong Xu, Haibo Wang, Jie Bao

**Affiliations:** 1 School of Mechanical and Materials Engineering, North China University of Technology, Beijing 100144, China; anneyan@126.com (Y.Y.); peace54321@126.com (J.B.); 2 College of Mechanical and Vehicle Engineering, Hunan University, Changsha 410082, China; xuhanzhong@hnu.edu.cn

**Keywords:** magnesium alloys, roll forming, springback, tension–compression asymmetry, anisotropy, yield criterion

## Abstract

Plastic forming in magnesium alloy sheet products is becoming a hot topic because of its potential in light-weight structural designs. Due to the special anisotropic and tension–compression asymmetrical properties of magnesium alloys, traditional modeling methods based on the von Mises yield criterion and using only uniaxial tensile properties for bending-dominated process simulations are not able to produce accurate predictions. In this study, two kinds of tensile tests (uniaxial and biaxial) and some compressive tests were performed along three material directions to obtain anisotropic and asymmetric properties, based on which the parameters of the Hill48 and Verma yield criteria were obtained. Then, the user subroutine VUMAT was developed, and the roll-forming process for magnesium alloys was simulated with the established anisotropic and asymmetric yield criteria. Finally, a roll-forming experiment on AZ31 magnesium alloy was performed. Compared with the experiments, it was found that roll-forming and springback predictions based on the Verma yield criterion had higher accuracy than those based on the von Mises and Hill48 yield criteria FEM models, which ignore anisotropy and asymmetry. This study provides an important FEM modeling idea that considers not only anisotropy but also asymmetry in the bending-dominated forming processes of magnesium alloys in which tension and compression exist simultaneously.

## 1. Introduction

Magnesium alloys are regarded as an effective light-weight solution in transportation industries and are receiving more and more attention, especially for spaceflight, motor vehicles, and high-speed rail, where light-weight materials are crucial [1,2]. Although the need for Mg alloys has risen dramatically, Mg alloy sheet-formed parts remain at a very low level in practical usage because, at room temperature, Mg alloys have obvious anisotropy and tension–compression asymmetry and limited formability [3].

Mg alloys are often bent to form large, curved structural components in order to improve aerodynamic performance in vehicles. Roll-forming technology is an essential forming process for metal sheets because it can produce many complex profiles, and it is widely used in major industries [4,5]. Roll forming has many advantages, including high efficiency, high accuracy, great surface quality, and high strength at low cost [6,7,8]. Aimed at forming Mg alloy parts, Mg alloy roll-forming technology has recently been proposed. In the automotive field, roll-formed Mg alloy profiles have potential applications in seat structures and car bodies [9].

Finite element analysis is becoming an important way to study different engineering problems under complex conditions. When sheet metal-forming processes are simulated in studies, yield criteria and constitutive models, which can describe the material’s anisotropic behavior, are fundamental [10]. The von Mises yield criterion [11] is the most famous and is sufficient to depict mechanical behaviors in isotropic metallic materials in general. However, it is not sufficient for describing anisotropic ones. In order to enable the yield criterion to explain anisotropy, Hill proposed an approximate quadratic criterion in 1948. The von Mises yield criterion was modified by bringing in several parameters [12,13]. Sheet metal-forming analysis has widely adopted the Hill48 yield criterion [14,15,16,17]. The Hill48 parameters can be solved with yield stresses or *r* in different directions [18]. Unfortunately, the anisotropic properties of Mg alloys are seldom considered in roll-forming and springback predictions, and ensuring roll-forming quality in production always depends on the specific manufacturing experience.

Mg alloys have been reported to have significant tension–compression asymmetry [19,20,21]. The initial yield stress of compression is obviously lower than the yield stress of tension. The microstructural mechanisms of the tension–compression asymmetry property have been explained in detail [22]. Firstly, it is due to the limited number of active slip systems at room temperature in hexagonal close-packed (HCP) alloys [3]. Secondly, it is due to the twinning mechanism that can contribute noticeably to plastic deformation [23,24,25,26,27]. This tension–compression asymmetry property will inevitably bring challenges to springback prediction in Mg alloy sheet roll-forming processes in which tension and compression exist simultaneously if the conventional FEM modeling method (only the tensile property is considered) is adopted.

The asymmetry property and its influences on bending have attracted the interest of many researchers. Zhou et al. [28] performed tension, compression, and bending tests to study how tension–compression asymmetry influences the radius of the curvature and energy absorption of Mg alloy sheets. Norbre et al. [29] performed a four-point bending test on AZ31B Mg alloy and observed the asymmetry by observing residual stresses. Emile et al. [30] also performed four-point bending tests on AZ31B. They measured the strain fields’ evolution and studied the tension–compression asymmetric behavior. Kurukuri et al. [19] performed tension and compression tests on AZ31B rolled sheets at variable strain rates and characterized the anisotropy and tension–compression asymmetry and the influence of variable strain rates on this behavior.

Some yield criteria have been proposed to describe the asymmetric yielding. Oana Cazacu [31] proposed an isotropic criterion that can depict the asymmetric yielding of pressure-insensitive metals. In addition, the isotropic criterion was extended to orthotropy. In textured Mg and Mg alloys sheets, the phenomenon that compressive strength is lower than tensile strength and tensile strength in the rolling direction is lower than in the transverse direction are both observed, which are reproduced with high accuracy by the proposed anisotropic yield criterion. Kim et al. [32] proposed a constitutive model to explain the anisotropy and asymmetry of AZ31B Mg alloy in a plane-stress state with the Cazacu isotropic yield criterion [33]. Through comparing an FEM simulation based on the developed constitutive models with experimental results for a three-point bending test, Verma et al. [34] observed that ultra-low carbon steel has tension–compression asymmetry, and they then modified the yield criterion proposed by Hill (1948) [35] and Hoffman (1967) [36] and proposed a non-symmetric yield criterion. Shi et al. [22] proposed some methods to describe the evolution of the subsequent yield loci. The present work investigates how the tension–compression asymmetry influences the springback in Mg alloy roll-forming process and establishes the FEM model incorporating anisotropic and asymmetric material models to predict the springback amount properly. Initially, quasi-static uniaxial tensile, biaxial tensile, and uniaxial compressive tests are performed to characterize the anisotropic and tension–compression asymmetric behavior. After that, the user subroutine VUMAT was developed to include the calibrated yield criterion that demonstrates the anisotropy and asymmetry. The FEM models of the Mg alloy roll forming considering both the anisotropy and the asymmetry are established. Finally, the roll-forming experiments were performed, and the corresponding FEM simulations are validated against the experimental results.

## 2. Materials and Methods

### 2.1. Uniaxial Tensile Tests

**Figure 1:**
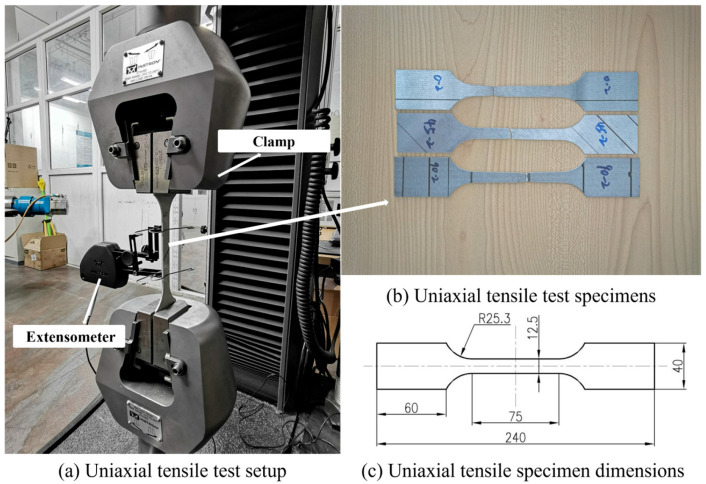
Uniaxial tensile test set up and the specimens after tests (unit: mm).

The uniaxial tensile tests were performed on a universal material testing machine, the INSTRON5980 (Instron, Norwood, MA, USA), as shown in Figure 1. The specimen preparation and testing procedure are carried out according to GB/T 228.1-2010 [37]. The test material is AZ31B Mg alloy sheet with a thickness of 2 mm. The specimens were prepared oriented in three directions—the rolling direction (RD), transverse direction (TD), and diagonal direction (DD), to study the anisotropy. Three sets of experimental tests were conducted for each directional orientation. Figure 1 illustrates the specimens used in the second set of these tests.

### 2.2. Uniaxial Compressive Tests

**Figure 2:**
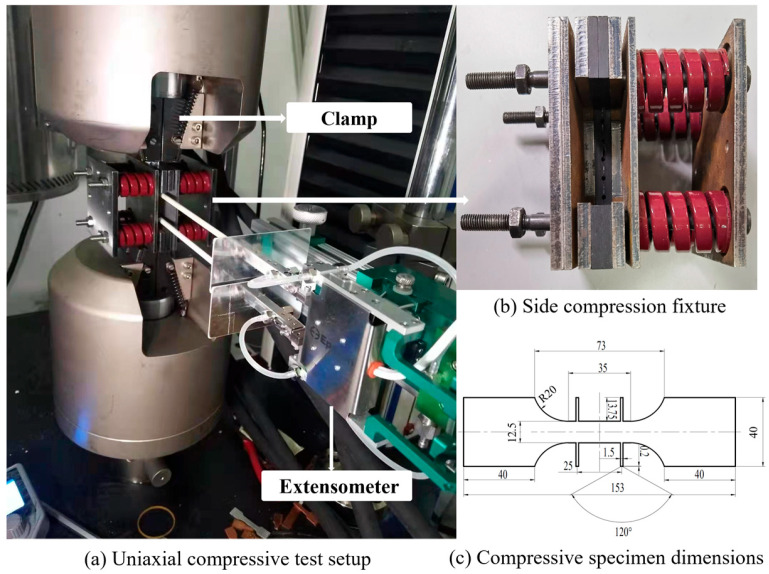
Side compression fixture in the uniaxial compressive test (unit: mm).

Some researchers have designed the side pressure fixtures to prevent the buckling on the specimens [38,39,40]. The idea of Dong et al. [41] is adopted where the anti-instability side pressure fixture is used, as shown in Figure 2, and a 3000 N compressive force was applied on the two sides of the specimen. The four extended ends on the specimen are used to attach the extensometer. Three sets of experimental tests in each directional orientation were conducted.

### 2.3. Biaxial Tensile Tests

**Figure 3:**
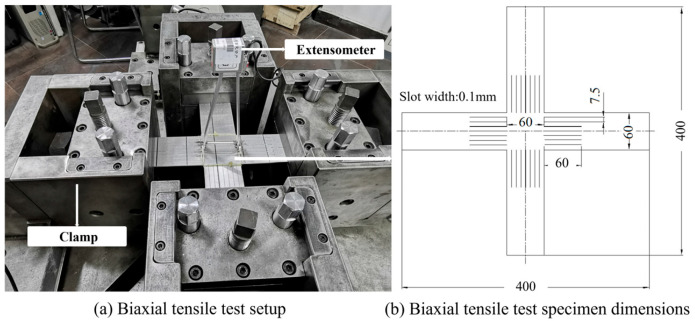
Variable load ratio biaxial tensile experiment (unit: mm).

The biaxial tensile tests were carried out on the variable ratio biaxial tensile machine. The established test setup is shown in Figure 3. Two extensometers are fixed on the upper and lower surfaces of the specimen, respectively, perpendicular to each other. The load ratio in the two directions is 1:1 to obtain the equal-biaxial tensile properties for the following calibration of Hill48 and Verma yield functions. The biaxial tensile specimens have thin slots along the four directions in order to obtain well-distributed strain in the central area (as shown in Figure 3). The cutting directions are along RD and TD, respectively.

### 2.4. Yield Stresses of AZ31B Obtained from Tensile and Compressive Tests

The initial yield stresses from uniaxial tensile, biaxial tensile and uniaxial compressive tests are shown in Table 1, in which T denotes tensile, C denotes compressive, and b denotes biaxial.

### 2.5. Roll-Forming Experiment

**Figure 4:**
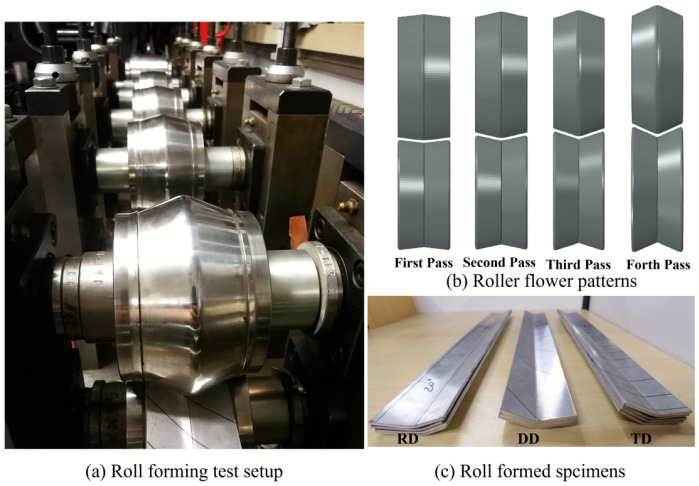
Roll-forming experiment setup and the formed parts.

A four-pass roll-forming experiment is carried out on an AZ31B metal sheet. The dimensions of the AZ31B sheet are 400 mm × 54 mm × 2 mm. The material directions of AZ31B Mg alloy in roll-forming experiments along the feeding direction are RD, DD, and TD, respectively. The roller stand distance is 250 mm. V-shaped rollers are adopted in this study. The bending angles are 5°, 10°, 15°, and 20°, respectively. The bending corner radii are 35 mm, 16 mm, 11 mm, and 10 mm, respectively, as shown in Figure 4.

### 2.6. Roll-Forming and Springback Process Simulations

In roll-forming processes, the plastic strain principally concentrates at the bending corner. It is known that the material at the outer surface is under a tensile state, and the inner surface is under a compressive state. As mentioned above, the AZ31B Mg alloy exhibits obvious tension–compression asymmetry. It not only influences the stress–strain relations but also strongly affects the yield surfaces. Therefore, if only the material properties from a uniaxial tensile (or compressive) test are adopted in simulations, as is widely used in FEM simulations of metal-forming processes, roll-forming springback prediction for AZ31B will inevitably exhibit considerable errors.

A simple method is to assign tensile and compressive properties to the outer section and the inner section of the bending corner. However, the neutral layer moves during the bending process. In other words, the tensile section and the compressive section change during the deformation process. In addition, if the objective cross-sections are complicated shapes like a “W” shape instead of a “V” shape, the simple method of assigning different material properties to different sections before deformation does not work well. Considering this problem, a user-defined material subroutine (VUMAT) has been developed to implement Verma yield criterion calibrated with tensile and compressive properties into ABAQUS/Explicit (https://www.3ds.com/products/simulia/abaqus, accessed on 18 February 2025).

The rollers are modeled as discrete rigid bodies, and the metal sheet is modeled as a deformable solid. Half of the roll-forming model is established with the symmetric boundary condition applied at the symmetry plane along the length direction. Set the friction coefficient to be 0.1 based on our previous Mg alloy roll-forming simulation experiences. The mesh size at the bending corner is 1.5 mm, while other parts of the sheet are 2 mm. Four layers of elements are adopted in the thickness direction. The rolling angular velocity ω is calculated and applied to each roller, ensuring the linear speed is constant through the whole length direction of the sheet.

## 3. Results and Discussion

### 3.1. Uniaxial Tensile Tests Analysis

**Figure 5:**
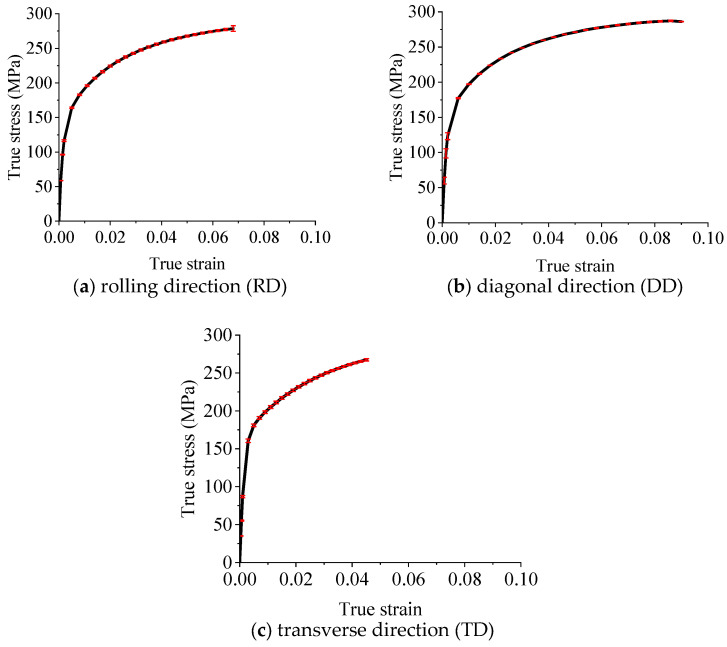
True stress–true strain curves of uniaxial tensile tests (the red symbols are error bars).

From the uniaxial tests shown in Figure 1, the curves of true stress and strain are obtained, as shown in Figure 5 where the error bars are also presented.

The narrow error bars in Figure 5 indicate a high degree of repeatability, suggesting that the material’s mechanical behaviors and properties are relatively stable, and the experimental conditions were well controlled and consistent. This level of precision demonstrates the reliability of the measurements. However, while error bars indicate precision, they do not account for systematic errors, such as calibration inaccuracies or environmental biases, which could affect the accuracy of the results. Therefore, the analysis of the tensile test results is carried out critically, and they are also compared with findings from other research articles as follows.

As depicted in Figure 5, the yield stress of the RD (rolling direction) is slightly lower compared to other directions, while the TD (transverse direction) exhibits the highest yield stress. Although the differences in yield stress are not significantly pronounced, this suggests a relatively minor in-plane tensile anisotropy. The variation in yield stress among different material directions is consistent with the findings of reference [32,42]. As to the yield stress values of the three directions, the findings of references [28,32,42] are also very close to the present study (yield strengths around 175 MPa).

The elongation characteristics of the materials vary considerably. Specifically, the DD (diagonal direction) demonstrates the maximum elongation, while the TD shows the minimum. The elongation results also require a comparative analysis. The observed variation in elongation among different material directions is consistent with the findings of [28,32,42] in terms of the elongation of the TD, which is obviously smaller than that of the other two directions. It can be found that the elongations of the RD and DD of these three studies are also different [28,32,42], among which the elongation order in the three directions of [42] is exactly the same as this study. However, our results show a more significant difference in elongation between DD and TD compared to what [28,32] documented, suggesting that our material may exhibit a unique combination of properties.

### 3.2. Uniaxial Compressive Tests Analysis

The uniaxial tensile test with side compression was conducted to eliminate the error caused by the friction force induced by the side compression fixture. The friction coefficient was calculated with Equation (1) [41].

(1) $$F=F_{0}-2(\mu F_{s})$$

where *F* is the crosshead load without the side compression in the uniaxial tensile test, *F*_0_ is the crosshead load with the side compression in the uniaxial tensile test, and *F_s_* is the side compression force.

**Figure 6:**
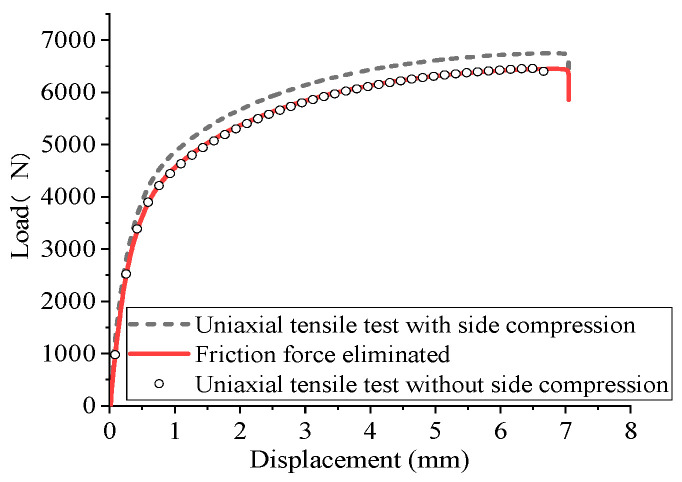
Original and friction force eliminated load–displacement comparisons.

The load–displacement relations of the uniaxial tensile tests with and without side compression are plotted with respect to the cross-head displacement, as shown in Figure 6. Forty friction coefficients from 0 to 2.0 have been tested to obtain the best friction coefficient value. It is found that when *µ* = 0.05, the load–displacement relation obtained by eliminating the friction force calculated with Equation (1) agrees well with the experimental result, as shown in Figure 6. Therefore, this friction coefficient can be used in the true stress calculation and FEM simulations of uniaxial compressive tests.

**Figure 7:**
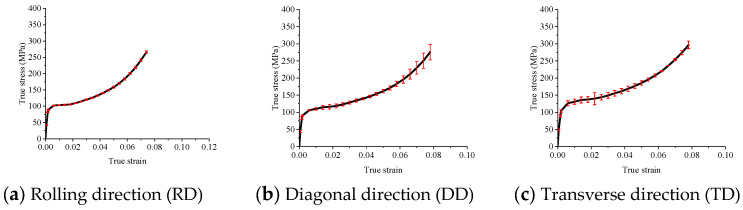
Stress–strain curves of uniaxial compressive test (the red symbols are error bars).

The true stress–strain curves obtained from uniaxial compressive tests along three distinct material directions are depicted in Figure 7. The experimental results reveal a significant anisotropy in the material’s mechanical response during the initial stage of plastic deformation. Notably, the true stress measured in the transverse direction (TD) is considerably higher than that observed in the other two directions. This phenomenon can be attributed to the crystallographic texture and preferred grain orientation developed during material processing, which enhances resistance to deformation along the TD. These findings offer valuable insights into the anisotropic deformation characteristics of the material and have important implications for its formability and mechanical performance in engineering applications. The error bars are also presented in Figure 7 further indicate the repeatability of the tests is good.

### 3.3. Hill48 Yield Criterion Under Plane Stress State

The material is mainly under plane stress conditions in sheet metal-forming processes. As the Hill48 yield criterion [35] mentioned earlier (Equation (2)), *x* is adopted as the RD and *y* as the TD.

(2) $$f=(G+H)\sigma_{x}^{2}-2H\sigma_{x}\sigma_{y}+(H+F)\sigma_{y}^{2}+2N\sigma_{xy}^{2}={\bar{\sigma }}^{2}$$

where $\sigma_{x}$ is the normal stress along the RD; $\sigma_{y}$ is the normal stress along the TD; $\sigma_{xy}$ is the shear stress; and *F*, *G*, *H* and *N* are parameters representing the anisotropy of the material.

Supposing that the RD is the reference direction, and choosing the yield stress $\sigma_{0}$ in the RD as the reference, then

(3) $$\sigma_{0}=\bar{\sigma }$$

Equation (4) can be derived from Equation (2):

*G* + *H* = 1 (4)

There are two methods which can be used to calibrate Hill48 yield criterion parameters:

(1) The Hill48 stress method based on the yield stress of different tension directions to calculate the parameters

(5) $$F=\frac{1}{2}\left({\left(\sigma_{0}/\sigma_{90}\right)}^{2}-1+{\left(\sigma_{0}/\sigma_{b}\right)}^{2}\right)$$

(6) $$G=\frac{1}{2}\left(1-{\left(\sigma_{0}/\sigma_{90}\right)}^{2}+{\left(\sigma_{0}/\sigma_{b}\right)}^{2}\right)$$

(7) $$H=\frac{1}{2}\left(1+{\left(\sigma_{0}/\sigma_{90}\right)}^{2}-{\left(\sigma_{0}/\sigma_{b}\right)}^{2}\right)$$

(8) $$N=\frac{1}{2}\left({\left(2\sigma_{0}/\sigma_{45}\right)}^{2}-{\left(\sigma_{0}/\sigma_{b}\right)}^{2}\right)$$

where $\sigma_{0}$, $\sigma_{90}$, $\sigma_{45}$ and $\sigma_{b}$ are the yield stresses of the RD, the TD, the DD and the equal biaxial tension, respectively.

(2) The Hill48–*r* method that through the *r* of different tension directions can be used to calculate the following parameters

(9) $$F=\frac{r_{0}}{(1+r_{0})r_{90}}$$

(10) $$G=\frac{1}{\left(1+r_{0}\right)}$$

(11) $$H=\frac{r_{0}}{\left(1+r_{0}\right)}$$

(12) $$N=\frac{\left(1+2r_{45})(r_{0}+r_{90}\right)}{2(1+r_{0})r_{90}}$$

where $r_{0}$, $r_{45}$ and $r_{90}$ are the *r* (strain ratios) under uniaxial tensile tests along the RD, the DD and the TD, respectively.

This study focuses on the springback prediction, so the requirements of the stress calculation accuracy is relatively high. Therefore, the stress method is used to determine Hill48 yield criterion parameters. Table 2 presents the anisotropic property parameters and the associated parameters needed in ABAQUS.

### 3.4. Verma Yield Criterion for Plane Stress Condition

The traditional Hill48 yield criterion cannot describe tension–compression asymmetry. Aiming to describe both the tension–compression asymmetry and the anisotropy of sheet metal simultaneously, Verma et al. [34] modified the Hill48 yield criterion by adding additional linear terms, as shown in Equation (13).

(13) $$a{\left\{\sigma_{x}^{2}-A\sigma_{x}\sigma_{y}+B\sigma_{y}^{2}+C\tau_{xy}^{2}\right\}}^{\frac{1}{2}}+\left(k_{1}\sigma_{x}+k_{2}\sigma_{y}\right)=\sigma_{0}$$

There are six parameters in the function, which can be calculated by using two tensile, two compressive yield stresses along the RD and TD ($\sigma_{0}^{T}$, $\sigma_{90}^{T}$, $\sigma_{0}^{C}$ and $\sigma_{90}^{C}$), a tensile yield stress along DD ($\sigma_{45}^{T}$) and the equal–biaxial tensile yield stress ($\sigma_{b}^{T}$). The six parameters are calculated with Equations (14)–(19).

(14) $$k_{1}=\frac{\sigma_{0}^{C}-\sigma_{0}^{T}}{2\sigma_{0}^{C}}$$

(15) $$k_{1}=\frac{\sigma_{0}^{C}-\sigma_{0}^{T}}{2\sigma_{0}^{C}}$$

(16) $$a=\frac{\sigma_{0}^{C}+\sigma_{0}^{T}}{2\sigma_{0}^{C}}$$

(17) $$B={\left\{\frac{\sigma_{0}^{C}\sigma_{0}^{T}\left(\sigma_{90}^{C}+\sigma_{90}^{T}\right)}{\sigma_{90}^{C}\sigma_{90}^{T}\left(\sigma_{0}^{C}+\sigma_{0}^{T}\right)}\right\}}^{2}$$

(18) $$A=1+B-{\left[\frac{1}{a}\left\{\frac{\sigma_{0}^{T}}{\sigma_{b}^{T}}-\left(k_{1}+k_{2}\right)\right\}\right]}^{2}$$

(19) $$C={\left[\frac{1}{a}\left\{\frac{2\sigma_{0}^{T}}{\sigma_{45}^{T}}-\left(k_{1}+k_{2}\right)\right\}\right]}^{2}-\left(1-A+B\right)$$

By substituting the yield stresses from Table 1 into Equations (14)–(19), the coefficients of Verma yield criterion can be calculated. Table 3 shows the parameters.

**Figure 8:**
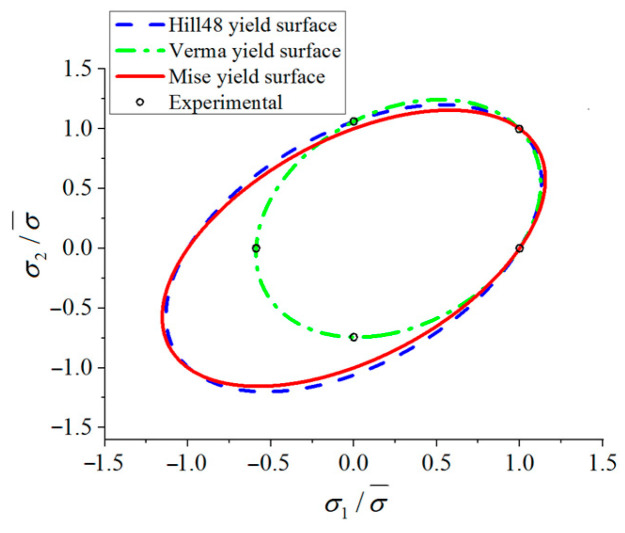
The von Mises, Hill48 and Verma yield surfaces.

The normalized von Mises, Hill 48 and Verma yield curves are compared in Figure 8, which shows that the tension–compression asymmetry has obvious influences on the yield curves. It can be seen that the upper right part of Verma yield curve is closer to the von Mises and Hill48 yield curves, while its lower left part is far away from the other two curves.

### 3.5. Roll Forming and Springback Analysis

**Figure 9:**
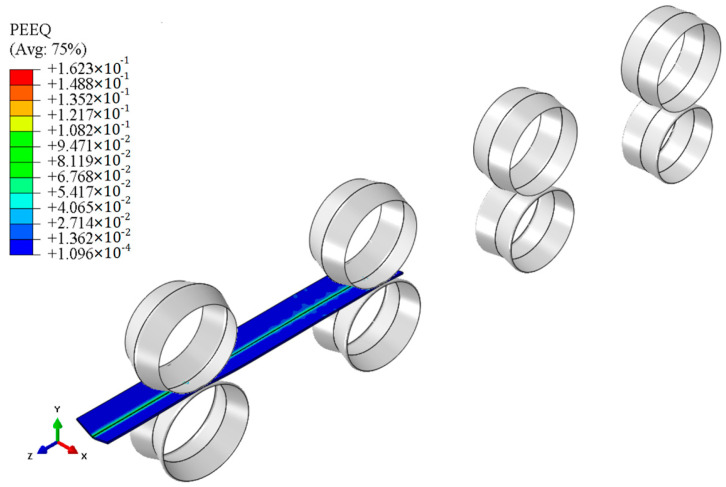
Plastic strain in roll-forming simulation.

**Figure 10:**
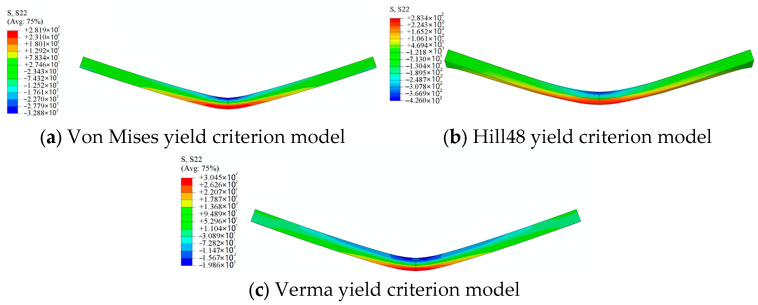
Bending stress along the width direction when passing through the last pair of rollers.

The plastic strain distribution of the specimen in the RD roll-forming simulation is shown in Figure 9. The tensile and compressive bending stresses along the width direction at the bending corners from simulations of the RD roll-forming experiment are shown in Figure 10.

**Figure 11:**
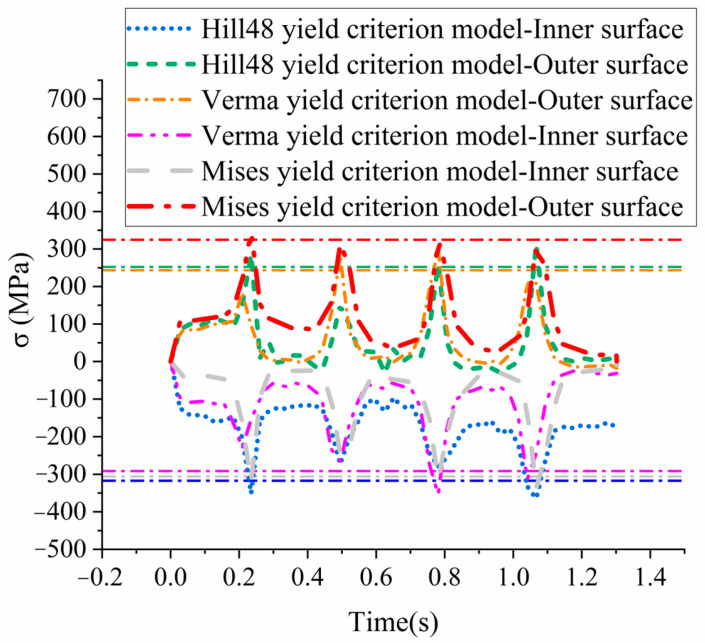
Bending stresses calculated with different material models.

Select one node from the inner surface and one node from the outer surface of the bending corner, and compare their bending stresses calculated with three yield criteria (von Mises, Hill48, and Verma yield criterion models), as shown in Figure 11. The average values of the peak stresses at the four bending passes are drawn as dashed lines in Figure 11, and they are listed in Table 4. It is shown that the tensile stresses at the outer surface of the Hill48 and Verma yield criterion models are relatively close, while the stresses of the von Mises yield criterion model are obviously higher than those of the other two models. As for the compressive stresses at the inner surface, it is shown that the stresses of the Hill48 and the von Mises yield criterion models are greater than that of the Verma yield criterion model. This perfectly proves that the tension–compression asymmetry obviously affects the calculation of the stresses in bending dominated forming processes, in which the tensile stresses and compressive stresses exist simultaneously. And the accuracy in the calculation of the stresses will then affect the accuracy of the springback prediction. The simulation results of the other two directions (TD and DD) are quite similar to those of the RD FEM models, so those results are not listed here.

**Figure 12:**
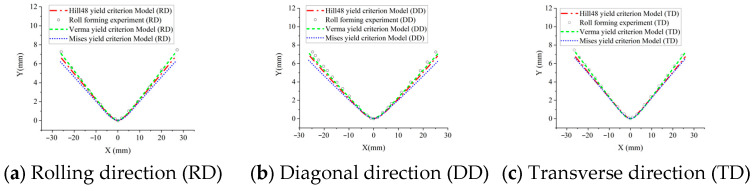
Cross-sections of roll-forming experiments and corresponding FEM simulations.

**Figure 13:**
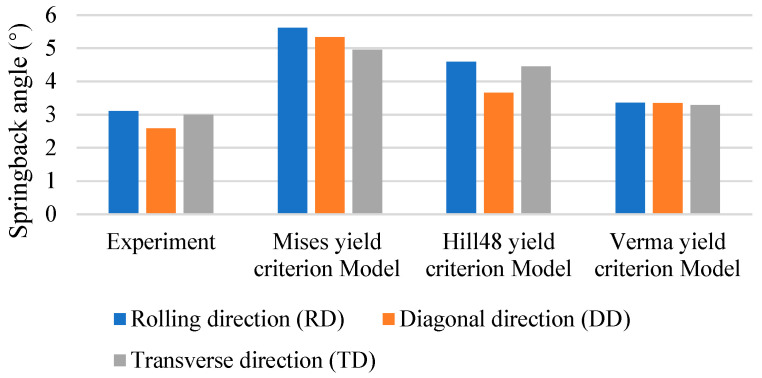
Springback angles from experiments and FEM simulations.

**Figure 14:**
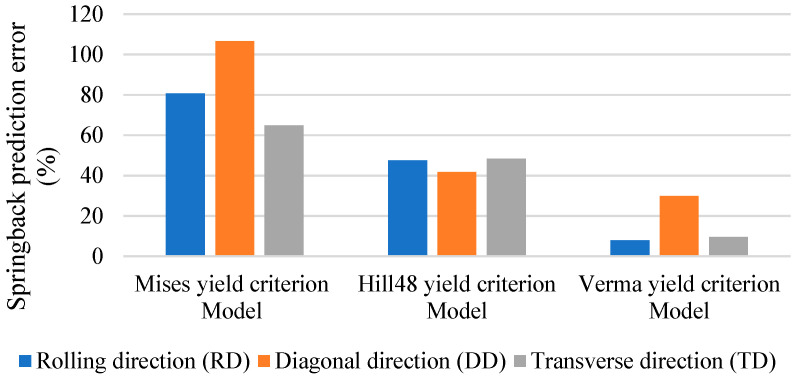
Springback angle prediction errors.

As shown in Figure 12, we compared the cross-sections of the experiments and three FEM models after roll forming and springback, respectively. The springback angles of the three models and the experiments are shown in Figure 13, and the calculated springback prediction errors are shown in Figure 14 and Table 5. It can be found that the springback prediction of the Verma yield criterion model exhibits higher accuracies than the von Mises and Hill48 yield criteria model. This improvement is due to the incorporation of the Verma yield criterion that accounts for the tension–compression asymmetry. Springback is a stress relaxation process, so the accurate stress calculation in the forming process strongly affects the springback prediction. Therefore, the proposed FEM model incorporating the Verma yield criterion is effective in predicting the bending dominated forming processes of Mg alloys. It is shown that for the calculation results of the Verma yield criterion FEM model, the error of the DD is the largest among the three material directions. This is because the DD compressive yield stress $\sigma_{45}^{C}$ was not used when calibrating the parameters of the Verma yield criterion, and only the DD tensile yield stress $\sigma_{45}^{T}$ is used, as shown in Equations (14)–(19).

In 2024, Wang et al. [43] also studied the springback of AZ31B magnesium alloy plates. They employed finite element simulation combined with experimental methods to perform the single-pass roll bending of AZ31B magnesium alloy plates at angles of 0°–15°, 0°–25°, and 0°–35°. Their calculated springback angles are 3.26°, 2.91°, and 2.16°, while the experimental springback angles are 2.44°, 2.07°, and 1.33°. Although the final bending angles are different, the springback cannot be compared directly; their springback prediction errors are 33.6%, 40.6%, and 62.4%, respectively, which are very similar to the errors calculated with the Hill48 yield criterion in this study. They employed the Hill48 yield criterion, but they did not consider the tensile–compressive asymmetry, which is possibly the reason for the springback prediction errors.

It can be seen from Equations (13)–(19) in Section 3.4 that the Verma yield criterion considers the tensile and compressive properties in the 0° and 90° directions, as well as the tensile properties in the equibiaxial tension and DD directions, totaling six material properties. These six material properties can be used to calibrate the six parameters of the Verma yield criterion. Compared with the Hill48 yield criterion, it considers the compressive properties in the 0° and 90° directions. For the roll forming studied in this paper, the main deformation area is in a bending deformation state, with compression on the inner side of the bending angle and tension on the outer side, and the amount of deformation in tension and compression is quite considerable. Since the AZ31B magnesium alloy studied in this paper has obvious tensile and compressive asymmetry, the Verma yield criterion, which considers both tension and compression, has high accuracy in predicting springback in roll forming, as mentioned above. Of course, the Verma yield criterion does not consider the compressive performance in the DD direction, so the prediction accuracy for roll forming in the DD direction is low. Fortunately, in real industrial production, there is no roll forming of materials in the DD direction. However, for other magnesium alloy plastic forming processes different from roll forming, if there is compressive deformation of materials in the direction around 45 degrees, the Verma yield criterion will no longer be applicable.

## 4. Conclusions

The FEM models comprehensively considering the anisotropic and the tension–compression asymmetric behavior of AZ31B magnesium alloy sheets in roll-forming springback prediction are established. With experimental and FEM simulation methods, the influence of tension–compression asymmetry on the roll forming springback is studied. There are some conclusions listed as follows:

(1) The uniaxial tensile tests, compressive tests and biaxial tensile tests of AZ31 magnesium alloy sheets were conducted along different material orientations. The results indicate that besides anisotropy, the material also exhibits obvious tension–compression asymmetry.
(2) A comprehensive comparative analysis of various yield criteria specifically for AZ31B magnesium alloy roll forming is carried out, which has not been extensively studied in this context. The parameters of the Hill48 anisotropic yield criterion (considering anisotropic properties) and the Verma yield criterion (considering both anisotropic and asymmetric properties) are calibrated based on the experimental results.
(3) So as to consider the tension–compression asymmetry in the FEM simulation of AZ31 Mg alloy roll-forming and springback process, the user-subroutine VUMAT including the established Verma yield criterion was developed.
(4) Compared with the von Mises and Hill48 yield criterion FEM models which ignore the tension–compression asymmetry, the final cross-sections calculated with the Verma yield criterion FEM model (Error: 8.04%) are much closer to the roll-forming experimental results than those from the von Mises yield criterion FEM model (80.7%) and Hill48 yield criterion FEM model (47.59%). This demonstrates that the proposed FEM modeling method based on the Verma yield criterion is reliable in magnesium alloy roll-forming and springback simulations. In roll forming, the tensile and compressive deformation amount is substantial. The results highlight the importance of considering tension–compression asymmetry in magnesium alloys roll forming for the first time, and they also emphasize the importance of adopting the proper yield criterion that can reflect the asymmetric yield behaviors in the magnesium alloy forming and springback predictions.

Future works include the characterization of the microstructure to link the asymmetric Verma yield criterion parameters to different grain distributions. The different microstructures developed during the cast and other manufacturing processes could impact the yield surface of Mg alloy sheets and can therefore explain potential differences with other studies. The comparison of residual stresses in formed Mg alloy sheets using non-destructive methods such as X-ray diffraction could also be conducted. These avenues are currently being pursued by the authors.

## Figures and Tables

**Table 1:** Yield stresses of AZ31B from tensile and compressive tests (unit: MPa).

$\sigma_{0}^{T}$	$\sigma_{45}^{T}$	$\sigma_{90}^{T}$	$\sigma_{b}^{T}$	$\sigma_{0}^{C}$	$\sigma_{45}^{C}$	$\sigma_{90}^{C}$
168.92	174.43	179.11	168. 50	99.43	104.19	125.61

Where $\sigma_{0}^{T}$ is tensile yield stress in RD, $\sigma_{45}^{T}$ is tensile yield stress in DD, $\sigma_{90}^{T}$ is tensile yield stress in TD, $\sigma_{b}^{T}$ is equal biaxial tensile yield stress, $\sigma_{0}^{C}$ is compressive yield stress in RD, $\sigma_{45}^{C}$ is compressive yield stress in DD, and $\sigma_{90}^{C}$ is compressive yield stress in TD.

**Table 2:** Anisotropic property parameters of AZ31B.

	*F*	*G*	*H*	*N*	*R*_11_	*R*_22_	*R*_33_	*R*_12_	*R*_13_	*R*_23_
Hill48	0.45	0.56	0.44	1.37	1	1.06	1.00	1.09	1.09	1.09

**Table 3:** Asymmetric anisotropic property parameters of AZ31B.

	*a*	*A*	*B*	*C*	*k*_1_	*k*_2_
Verma	1.349	0.395	0.719	2.073	0.349	0.201

**Table 4:** Average stresses at the bending corners from different FEM models.

	Hill48 Inner	Hill48 Outer	Verma Inner	Verma Outer	Von Mises Inner	Von Mises Outer
Average bending stress (MPa)	−317.43	252.07	−291.5	243.3	−306.12	324.4

**Table 5:** Springback angle prediction errors based on different FEM methods.

	Experiment (°)	Von Mises Yield Criterion Model Error (%)	Hill48 Yield Criterion Model Error (%)	Verma Yield Criterion Model Error (%)
Rolling direction (RD)	3.11	80.7	47.59	8.04
Diagonal direction (DD)	2.58	106.6	41.86	29.84
Transverse direction (TD)	3.00	65.0	48.33	9.67

## Data Availability

The original contributions presented in the study are included in the article, further inquiries can be directed to the corresponding author.
